# Poison Romance and Poison Mysteries

**Published:** 1900-03

**Authors:** 


**Poison Romance and Poison Mysteries.** By C. J. S. Thompson. Pp. viii., 255. London: The Scientific Press, Ld. 1899.

Mr. Thompson deals with a mysterious subject, one which always attracts a large number of readers, guided often by intelligence, but much more largely by curiosity. The work is clearly and well written, but is evidently intended for the lay rather than the professional mind. The "Poisons of Antiquity" go back into the remote ages. "Gula" seems to have been the patroness of medicine and a divinity of the Arcadians, as far back as 5000 B.C. In the more classical times poisons were looked upon as a luxury, and only the wealthy could ensure a safe and prompt dispatch, and certain professional miscreants had their tariff of charges in accordance with the social rank of their intended victims. No doubt several of the most ancient active poisons were ptomaines combined with arsenic or other ingredients. Nine chapters—vii. to xv. inclusive—relate the histories of as many celebrated criminals—between the years 1840 and 1889, or from the case of Mme. Lafarge to Mrs. Maybrick.

In a book on "Poison Romance" something, amongst general questions, might have been said about the charges made against the Jews of poisoning the wells in the times of the Black Death; and among particular instances, there might have been some reference to the alleged attempts at poisoning Queen Elizabeth by Dr. Lopez, who is supposed by some authorities to have been taken by Shakspere for his portrait of Shylock. Local pride also looked for the inclusion of Mr. William Herapath's name in the record of "The Rugeley Mystery." Mr. Thompson, by the way, seems to be unaware that Shakspere took the details of Juliet's potion direct from Arthur Brooke's translation of Bandello's version of the story.

We can recommend the book to anyone interested in these terrible crimes, and who is not? Why is there no index?

